# Mesenteric Vasculitis and Urinary System Involvement Presenting As the Initial Manifestations of Systemic Lupus Erythematosus Treated Successfully With Glucocorticoids and Rituximab: A Case Report

**DOI:** 10.7759/cureus.31474

**Published:** 2022-11-14

**Authors:** Roaa Alsolaimani

**Affiliations:** 1 Internal Medicine, King Abdulaziz University, Jeddah, SAU; 2 Internal Medicine, King Faisal Specialist Hospital and Research Centre, Jeddah, SAU

**Keywords:** abdominal pain, systemic lupus erythematosus, rituximab, lupus mesenteric vasculitis, gastrointestinal vasculitis

## Abstract

We describe a case of systemic lupus erythematosus (SLE) presenting initially with mesenteric vasculitis and urinary system involvement without any other SLE clinical features and a weakly positive antinuclear antibody on initial testing. She is a 15-year-old healthy female who presented with severe abdominal pain, diarrhea, vomiting, and weight loss for more than one month. She later developed cystitis and bilateral hydronephrosis. Her repeated autoantibody panel and computed tomography of the abdomen were diagnostic for mesenteric vasculitis related to SLE, and a colonic biopsy showed occlusive thromboangiopathy related to associated antiphospholipid antibody syndrome. She responded well to intravenous methylprednisolone, rituximab, and anticoagulation. Mycophenolate mofetil and hydroxychloroquine were used later for maintenance treatment.

Lupus mesenteric vasculitis (LMV) is rare but a serious, potentially life-threatening, gastrointestinal complication associated with SLE. Clinical features are not very specific, making them challenging to diagnose. Thus, early diagnosis requires a high index of suspension especially in cases presenting with gastrointestinal symptoms without a previously established diagnosis of SLE. LMV occurs as an initial presentation of SLE in children more than adults. So, it is wise to investigate children presenting with gastrointestinal manifestations and involvement of other organs especially the urinary system thoroughly with autoantibodies and abdominal imaging to rule out or confirm the diagnosis of LMV and to start treatment early and aggressively.

## Introduction

Gastrointestinal manifestations are common in systemic lupus erythematosus (SLE). They vary from minor symptoms such as oral ulcers and nausea to major manifestations such as hepatitis, peritonitis, pancreatitis, and mesenteric vasculitis. A recently published systematic review by Frittoli, et al. [1] summarizes the gastrointestinal involvement patterns in patients with SLE.

Mesenteric vasculitis is an uncommon gastrointestinal complication in SLE patients, with a reported prevalence ranging from 0.2 to 9.7% [2,3]. In a retrospective study, 97 out of 3823 patients with SLE were diagnosed with Lupus mesenteric vasculitis (LMV) with an overall prevalence of 2.5% in that cohort [4].

LMV usually occurs in patients with a long-standing history of SLE or in patients with high disease activity and usually affects the small intestine (jejunum and the ilium) [3]. It rarely occurs as the initial manifestation of SLE like the case we are describing and a few others reported in the literature [5,6].

We report a case of a 15-year-old female, not known to have SLE, presenting with LMV without other SLE features. The diagnosis of SLE was later confirmed with a positive ANA, low complement levels, positive antiphospholipid antibodies, and a colonic biopsy confirming occlusive thromboangiopathy. This is the second case to be reported in Saudi Arabia [6].

## Case presentation

A 15-year-old female presented to the emergency department with abdominal pain, persistent vomiting, and non-bloody diarrhea (more than 10 times per day) for two months. These symptoms were associated with anorexia and weight loss of 10 kilograms (kg). She denied fever, night sweats, contact with sick or tuberculosis patients, recent travel, or raw milk ingestion. Her symptoms started shortly after eating a contaminated meal. She denied any history of arthritis, malar rash, photosensitivity, alopecia, sicca symptoms, oral ulcers, genital ulcers, or Raynaud’s. She denied any neurological or cardio-respiratory symptoms. She gave a history of spontaneous bruises and mild intermittent epistaxis for one year. Over the past two months, she had multiple Emergency department visits with enteritis symptoms which were managed with supportive intravenous fluids, anti-emetics, and antibiotics. She was admitted once (one month prior to presentation) in a different facility with similar symptoms in addition to massive ascites. during that admission, Seven liters were drained from her abdomen. Computed tomography (CT) of the abdomen revealed thickening in the stomach, proximal jejunum, and scattered lymphadenopathy. Upper endoscopy showed gastritis and the biopsy was positive for H. pylori for which she was started on appropriate treatment. Stool culture was positive for Entamoeba histolytica. At the time they also noted thrombocytopenia (70x109/L), for which she was started on corticosteroids (prednisone 1mg/kg once daily) for possible idiopathic thrombocytopenic purpura (ITP). Her abdominal pain, appetite, and diarrhea improved dramatically as well as her platelet counts on prednisone. She was then discharged from the hospital with a tapering prednisone dose. She was doing well, and her diarrhea resolved, but symptoms recurred whenever the prednisone dose drops below 20 mg daily, for which she presented to the hospital and was admitted to our tertiary care hospital.

She was recently diagnosed with hypothyroidism and was on levothyroxine. she has no other relevant medical or surgical history. She is a high school student who lives with her parents. Her aunt has SLE.

Upon admission, she was conscious and oriented. She was underweight and dehydrated. She was afebrile, with a heart rate of 100 beats/minute, and a blood pressure of 125/75 millimeters of mercury with a postural drop. She had no skin rash, oral ulcers, arthritis, or palpable lymphadenopathy. Her chest, cardiovascular and neurological examinations were normal. Her abdomen was soft, with diffuse tenderness but more tender at the left lower quadrant and epigastric area with no rebound or rigidity.

Her initial laboratory investigations showed leukopenia: 3.0x109/L, hemoglobin level: 130 g/L, thrombocytopenia: 51x109/L, normal creatinine, low potassium, normal liver function except for low albumin, normal coagulation profile and a normal TSH. She had a normal Chest x-ray and echocardiogram.

She was initially managed with intravenous fluids, anti-emetics, and intravenous hydrocortisone (25 mg every eight hours). Other investigations including full septic screen, quantiFERON-TB, stool cultures including C. difficile culture, celiac antibodies, and fecal calprotectin were ordered. In addition to peripheral blood film, vitamin B12, iron, folate, and viral serology (hepatitis, parvovirus, EBV, dengue). All the above investigation results came back normal. An autoantibody panel including antinuclear antibody (ANA), Anti-dsDNA, and antiphospholipid antibodies (APLA) was also ordered.

On the second day of hospitalization, a CT scan of the abdomen was performed and revealed descending and recto-sigmoid colon thickening suggestive of inflammatory bowel disease (IBD). Upper endoscopy showed edematous gastric mucosa, narrowing between the second and third part of the duodenum and possible thickening in the jejunum. Her colonoscopy was completely unremarkable. Biopsies from the stomach, duodenum, terminal ileum, and colon were unremarkable. MRI enterography showed inflammatory bowel wall thickening from the left colon to the rectum and to a lesser extent in the proximal jejunum. After a few days and while receiving hydrocortisone, her abdominal pain and diarrhea improved.

The gastroenterology and infectious disease teams excluded IBD or any possible infectious causes including intestinal tuberculosis based on negative imaging, cultures, and normal biopsies. She was seen by the rheumatology team after the ANA titer result came back weakly positive (1:40). Her repeated laboratory workup revealed worsening leukopenia: 2.25 x109/L, with lymphopenia 0.39 k/uL, and improved platelets counts: 118x109/L. Otherwise, normal C-reactive protein (CRP), Normal renal function, and urine analysis. She had a negative anti-dsDNA, anti-Ro, anti-La, anti-smith, and anti-RNP. On the other hand, she had a positive anticardiolipin IgM (22 MPL/ml, reference range 0.0-12) and B2-glycoprotein IgM (28 SM U/ml, reference range 0.0-20) antibodies, positive direct monospecific coombs test and low complements ( C3: 0.67 g/L, reference range 0.90-1.80 and C4: 0.07 g/L, reference range 0.10-0.40). 

**Figure 1:**
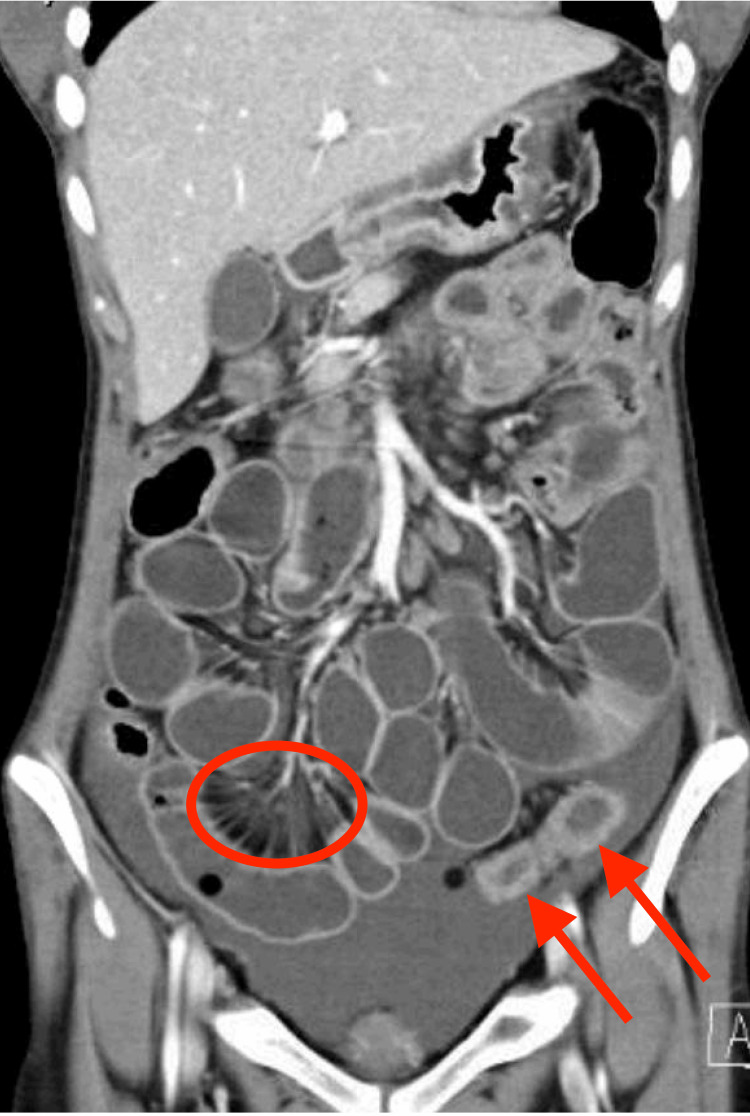
Sagittal contrast enhanced CT scan of the abdomen and pelvic showing circumferential small bowl wall thickening and edema “arrows” with prominent engorgement of mesenteric vessels (comb sign “circle”).

Over the course of one month, she was gradually improving. Her corticosteroids were changed from hydrocortisone to oral prednisone (25 mg daily). Her diarrhea had stopped completely but she felt weak and was not fully tolerating oral intake. The platelets and white cell counts also improved. On hospital day 32, she developed acute abdominal pain with guarding, so an urgent CT abdomen was done showing multiple dilated distal ileal loops representing ileus, abnormal identification of the superior mesenteric artery at the duodenojejunal junction likely related to superior mesenteric artery syndrome, increased ascites with new moderate bilateral hydroureteronephrosis. In addition to persistent abnormal wall thickening and edema of the sigmoid colon and proximal jejunum. These changes were consistent with mesenteric vasculitis [Figure 1].

She was immediately evaluated by the surgical team who recommended conservative treatment. She was also evaluated by the urology team who attributed the hydroureteronephrosis to the grossly dilated colon and ascites leading to obstruction and recommended no intervention and to repeat images after bowel decompression. She developed new cystitis symptoms confirmed by a positive urine culture for which she was started on antibiotics. 

**Figure 2:**
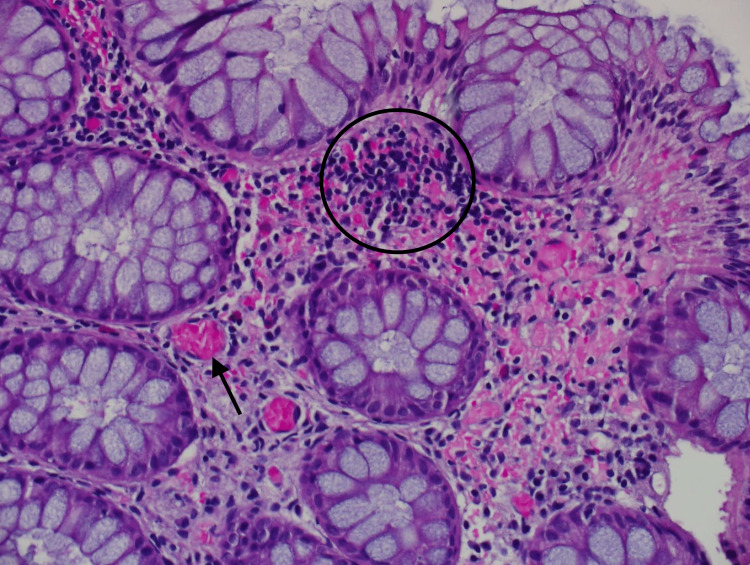
Tissue biopsy from the sigmoid colon illustrating focal small blood vessels occlusive micro-thrombi “arrows” and fibrin deposition “circle”. In addition to patchy distribution of fragmented red blood cells with extravasation in the lamina propria.

The presence of persistent abdominal pain with dilated bowel loops, intestinal wall thickening, bilateral hydronephrosis, and cystitis, lead us to repeat colonoscopy and biopsy. The laboratory investigations were suggestive of SLE, including low complements (C3 and C4), positive coombs test, and positive anticardiolipin and B2 glycoprotein antibodies. In addition to a repeated strongly positive ANA test (titer 1:320). Her colonic mucosal biopsy showed focal small blood vessels occlusive micro-thrombi and fibrin deposition. In addition to the patchy distribution of fragmented red blood cells with extravasation in the lamina propria [Figure 2]. The biopsy was diagnostic for antiphospholipid syndrome (APLS).

The diagnosis of SLE and APLS with gastrointestinal vasculitis and genitourinary involvement was confirmed. Intravenous pulse methylprednisolone (500 mg) was given for three days then shifted to oral prednisone (1 mg/kg once daily) with a gradual taper. She was also started on rituximab (375 mg/m2 weekly for four consecutive weeks), in addition to therapeutic anticoagulation using enoxaparin. The patient's symptoms improved dramatically within a few days and were able to tolerate orally gradually and was discharged home after the first rituximab infusion. She was then seen weekly for rituximab infusions and in the clinic afterward. She continued to improve clinically and was started on mycophenolate mofetil in addition to hydroxychloroquine as a maintenance medication for SLE. She is being followed in the clinic on a regular basis and continues to do well until her last visit four years after her initial presentation and diagnosis. Of note, her repeated anti-dsDNA came back positive (573 IU/ml, reference range0.0-200) at some point during the follow-up period and her repeated antiphospholipid antibodies were still positive 12 weeks after the initial result confirming APLS.

## Discussion

This report reviews a unique and challenging case of LMV. Some reports suggested that gastrointestinal infections can predispose to LMV recurrence but it is not clear whether it can be the trigger for the first presentation. Our patient had a history suggestive of food poisoning and a positive stool culture for Entameoba prior to her illness. LMV was her initial presentation of SLE which is very rare but reported more in children [7]. Our patient reported epistaxis and bruises for a year prior to presentation which are likely due to chronic thrombocytopenia that was not investigated. She also developed cystitis and bilateral hydronephrosis at a later stage of her presentation. Urinary involvement is frequently seen with LMV and is secondary to elevated anti-smooth muscle antibodies which can damage the smooth muscles in the intestine and urinary system. Other than thrombocytopenia, she had no other typical SLE features. Her ANA result was not impressive initially which is difficult to explain but repeated titer was strongly positive in addition to a positive anti-dsDNA later during the follow-up period. Similar reported cases showed moderate to high SLE disease activity scores measured by the SLE disease activity index (SLEDAI) on initial SLE presentation with LMV [5,8]. Our patient had a SLEDAI score of 12 points for the presence of vasculitis, low complements, leukopenia, and thrombocytopenia. Her SLEDAI score indicated high disease activity. 

Her first biopsies from the upper and lower endoscopies were negative for any pathology. This is not surprising, previous reports showed that most histopathologic samples taken by endoscopy were superficial and could not yield a definite diagnosis of LMV despite the presence of immunological parameters of the disease [2]. Post-operative surgical biopsies are more diagnostic. Furthermore, she didn’t have a jejunal biopsy, which is a more affected site in this disease in addition to the terminal ilium compared to the stomach and rectum. Also, she presented to our hospital on prednisone (20mg equal to 0.5mg/kg) for thrombocytopenia which could have partially helped her symptoms but at the same time delayed the proper diagnosis of LMV.

When her laboratory workup was completed in addition to her CT abdomen findings that were diagnostic for vasculitis and repeated colonic biopsy showing evidence of thrombotic microangiopathy suggestive of associated APLS, her lupus diagnosis was confirmed. She was classified to have SLE based on the Systemic Lupus Erythematosus International Collaborating Clinics (SLICC) classification criteria.

Lupus mesenteric vasculitis (LMV) is a rare gastrointestinal complication in SLE patients with a reported prevalence ranging from 0.2 to 9.7% and from 29% to 65% in SLE patients presenting with acute abdominal pain [2,3,9]. LMV is challenging to diagnose especially in the absence of typical SLE features. LMV is the most common cause of acute abdominal pain in children with SLE. Children were admitted more often due to LMV than adults with SLE (31.6% versus 13.9%) and had more recurrent episodes [10]. LMV is serious with a high mortality rate if misdiagnosed and mistreated. It may lead to further complications including intestinal hemorrhage, infarction, and perforation. Mortality was reported as high as 13.4% in the Chinese cohort [4]. Thus, It is very important to make the proper diagnosis of LMV early. LMV symptoms are usually non-specific and variable but the most common symptoms are abdominal pain, tenderness, and bleeding per rectum, with the presence of nausea, vomiting, and diarrhea [11]. CT abdomen is needed to diagnose LMV. The most common imaging findings are engorgement of mesenteric vessels (comb sign), increased attenuation of mesenteric fat, bowel wall edema and thickening, dilatation of intestinal segments and target sign [2,12], In addition to ascites and urinary tract involvement. Histopathologic confirmation may be difficult but should be obtained when feasible.

The first line of treatment for LMV is corticosteroids. The disease often shows an excellent response [13,14]. Additional immunosuppressive medications such as cyclophosphamide, mycophenolate mofetil, or rituximab may be added in cases with severe disease, other organ involvement, or in refractory cases [4,12,15]. We chose to give our patient rituximab early in conjunction with corticosteroids given her severe and prolonged disease course and involvement of the urinary symptoms. 

## Conclusions

Gastrointestinal vasculitis and cystitis can be the initial manifestations of SLE. They can be associated with morbidity and mortality and require a high index of suspicion for timely diagnosis. Investigate children presenting with gastrointestinal manifestations and involvement of other organs especially the urinary system thoroughly with autoantibodies and CT abdomen to confirm the diagnosis of LMV. Treat with corticosteroids and add immunosuppressive medications in severe or refractory diseases.
